# Dr. Thomas Graham Wier’s Communication Illustrating the Durability of the Vaccine Virus

**Published:** 1842-08-27

**Authors:** Thomas Graham Weir


*Communication illustrating the durability of the Vaccine Virus, read before the Edinburgh Medico-Chirurgical Society.* By THOMAS GRAHAM WEIR, M. D.—In the year 1842, my friend, Dr. William Scott, was appointed Surgeon to the Honorable East India Company's Ship, the Marchioness of Ely, bound for India, having on board a detachment of the 16th Lancers, and a number of women and children. Small-pox being at this time prevalent in London and in its neighborhood, Dr. Scott, (apprehensive that if might break out on board,) requested a supply of vaccine virus from the London Vaccine Institution.

Four charges preserved in glass tubes, and eight between two pieces of glass, wrapped round with gold-beater's skin, were sent him.

No appearance of small-pox having occurred during this voyage nor during other three made by Dr. Scott to the same place, the vaccine virus remained unopened in his sea chest till March 1st, 1842. When wishing to ascertain the efficacy of the plan adopted by the National Vaccine Institution for preserving and transporting vaccine virus, he requested me to try it.

In accordance with his wish, on Friday the 25th of March 1824, I vaccinated two children at the Edinburgh New Town Dispensary with the virus contained in the glass tubes.

The plan adopted was the following; The small extremity of each tube having been broken off, the heat of the finger and thumb applied to the bulb was sufficient to expel the enclosed virus, which, being received upon the lancet, was carefully painted in, drawing as little blood as possible.

Friday, the 1st of April, eight days after vaccination, both children returned, when I found four vesicles as perfect as those done with vaccine virus three days old. Upon puncturing the vesicles, I obtained two full charges of vaccine lymph, which I sent to Dr. Hue, Registrar to the London National Vaccine Institution, from whom I received the following reply.

"Sir,—I have much pleasure in informing you, that the lymph you was so good as to send to us has been tried by order of the Board, and has fully succeeded. I am desired to thank you for your very interesting communication. The fact is highly important and deserves to be made known.

I am, Sir, C. Hue, Registrar."

Encouraged by the above success, I tried the lymph preserved between the two pieces of glass.

Three children having been brought for vaccination to the Edinburgh New Town Dispensary on Tuesday, the 14th of May, I removed the vaccide lymph from two of the charges, and employed them to make one vesicle on each child, at the same time making in each a second vesicle with fresh vaccine virus. Two returned on Tuesday the 21st of May, when I found in one two vesicles similar in size, and equally full of lymph. In the other, the vesicle formed from the old matter was smaller than that formed from the fresh virus, but quite as perfect, and containing a considerable quantity of vaccine lymph, some of which I employed with perfect success.—*Ibid.*

